# Research on the Food Security Condition and Food Supply Capacity of Egypt

**DOI:** 10.1155/2014/405924

**Published:** 2014-03-04

**Authors:** Jian Deng, Youzhen Xiang, Wenhui Hao, Yongzhong Feng, Gaihe Yang, Guangxin Ren, Xinhui Han

**Affiliations:** ^1^College of Agronomy, Northwest A&F University, P.O. Box 95, Shaanxi 712100, China; ^2^The Research Center of Recycle Agricultural Engineering and Technology of Shaanxi Province, Yangling, Shaanxi 712100, China; ^3^College of Water Resources and Architectural Engineering, Northwest A&F University, Yangling, Shaanxi 712100, China

## Abstract

Food security is chronically guaranteed in Egypt because of the food subsidy policy of the country. However, the increasing Egyptian population is straining the food supply. To study changes in Egyptian food security and future food supply capacity, we analysed the historical grain production, yield per unit, grain-cultivated area, and per capita grain possession of Egypt. The GM (1,1) model of the grey system was used to predict the future population. Thereafter, the result was combined with scenario analysis to forecast the grain possession and population carrying capacity of Egypt under different scenarios. Results show that the increasing population and limitations in cultivated land will strain Egyptian food security. Only in high cultivated areas and high grain yield scenarios before 2020, or in high cultivated areas and mid grain yield scenarios before 2015, can food supply be basically satisfied (assurance rate ≥ 80%) under a standard of 400 kg per capita. Population carrying capacity in 2030 is between 51.45 and 89.35 million. Thus, we propose the use of advanced technologies in agriculture and the adjustment of plant structure and cropping systems to improve land utilization efficiency. Furthermore, urbanization and other uses of cultivated land should be strictly controlled to ensure the planting of grains.

## 1. Introduction 

Food security is one of the most important issues in the world. The World Food Summit (1996) defines food security as a situation wherein all people at all times have physical, social, and economic access to sufficient, safe, and nutritious food that meets dietary needs and food preferences for an active and healthy life [1]. The UN Food and Agriculture Organization (FAO) estimates that 1.35 billion people around the world have insufficient food, 0.87 billion people are chronically hungry, and nearly 0.2 billion children are hypogenetic because of malnutrition [2]. The effects of this predicament may echo throughout generations. Global population is considered to continue to increase and may reach 9 billion in the middle of this century [3]. Therefore, food security, resource contention, environmental disruption, and other issues will also increase. The ever-evolving food price crisis has been a warning call for global food security in recent years. According to the official statistics of FAO, the global food price index rose 61% and wheat rice prices increased rapidly from June 2007 to March 2008. Although grain production has been increasing, 21 African countries (1/3 of the total number of African countries) are still included in the list of countries that experience food shortages and need outside help [4].

Arab Republic of Egypt is located in northeast Africa between latitudes 22°−32°N and longitudes 25°–34°E. Egypt has the largest land area (1,001,450 km^2^) and population in Africa. Egypt is considered as a heavily populated country with approximately 80 million (in 2011) people living on 4% of the land. The major agricultural region of Egypt is spread along the Nile Valley and Nile Delta [5]. Approximately 96% of the land is covered by deserts (e.g., Libyan Desert) with scarce precipitation, thus making farming difficult. Agriculture was made the pillar industry in the national economy of Egypt to ensure food security. However, global climate change, soil desertification, salinization, urbanization, and other issues have negatively affected grain production in Egypt [6]. Increasing country populations and fluctuating global food prices have increased the severity of food security problems. Improvements in food security are useful to reduce hunger and poverty and to promote economic development. Egypt is a representative country on global food security research because Egypt has implemented a food subsidy policy for more than 50 years and is a grain importing country [7]. Research on the Egyptian food security situation is significant to understand the current food supply situation in Africa and the world.

Previous research in Egyptian food security has mainly focused on food subsidy policies and changes in arable land by using mathematical models and analyses. Löfgren and El-Said used a computable general equilibrium model to analyse alternative options for the food subsidy system of Egypt [8]. Ahmed and Bouis provide a process of moving the optimal income-predicting model to the final mode by mainly aiming at poor households [9]. Ramadan and Thomas evaluate the impact of a reform in Egyptian subsidy policy on consumer demand for food and on consumer welfare by using the mixed demand model [7]. Wichelns describes an advantageous application, that is, “virtual water,” which is considered a key factor of production [10]. The study of cultivated land mainly focuses on land reclamation and soil salinization [5, 11–13].

(1) Our study comprehensively analysed the historical and current states of food security by using grain production condition data from 1960 to 2010; (2) it used the grey system model and scenario analysis to predict the future Egyptian population and to forecast future grain production conditions, respectively; (3) the results were combined to analyse the development tendency of grain production, food supply capacity, and population carrying capacity; (4) thereafter, several feasible development countermeasures have been proposed.

## 2. Data and Methodology

### 2.1. Grey System Analysis and GM (1,1) Model

Grey system theory (GST) was presented by Ju-Long [14]. The object of the grey system is partially unknown, uncertain, or incomplete system. This object requires only a limited knowledge and understanding of an unascertained system to solve problems, conduct good estimations, or perform predictions [15].

The model sign of the grey system is GM (*n*, *h*), where *n* is the order of the differential equation and *h* is the number of variables. One order and one variable exist in ordinary research; that is, *n* = 1 and *h* = 1. Thus, we obtain the GM (1,1) model. This model is the most important part in GST, which is widely used in time series forecasts and is renewed as new data become available to the prediction model [16]. The GM (1,1) model has been applied in online population and areal population forecasts [17–19].

The steps in conducting forecasts based on the GM (1,1) model can be referenced in the studies of Kayacan et al., Ju-Long, and Sifeng et al. [16, 20, 21]. We used the GM (1,1) model to conduct predictions on the Egyptian population. The brief steps of the prediction are presented as follows.

(1) Set up a time sequence *X* ^(0)^,
  (1) $$\begin{array}{c} X^{\left(0\right)}=\left(x^{\left(0\right)}\left(1\right),x^{\left(0\right)}\left(2\right),\ldots ,x^{\left(0\right)}\left(n\right)\right). \end{array}$$
  When sequence *X* ^(0)^ is subjected to the accumulating generation operation, the following sequence *X* ^(1)^ is obtained:
  (2) $$\begin{array}{c} X^{\left(0\right)}=\left(x^{\left(0\right)}\left(1\right),x^{\left(0\right)}\left(2\right),\ldots ,x^{\left(0\right)}\left(n\right)\right), \end{array}$$
  where
  (3) $$\begin{array}{c} x^{\left(1\right)}\left(k\right)=\sum_{i=1}^{k}x^{\left(0\right)}\left(i\right)=x^{\left(1\right)}\left(k-1\right)+x^{\left(0\right)}\left(k\right). \end{array}$$
(2) The whitening differential equation can be obtained from sequence *X* ^(1)^,
  (4) $$\begin{array}{c} \frac{{dx}^{\left(1\right)}\left(t\right)}{dt}-ax^{\left(1\right)}\left(t\right)=b. \end{array}$$
(3) According to (4), the solution of *X* ^(1)^ (*t*) at time *k* can be expressed as follows:
  (5) $$\begin{array}{c} x^{\left(1\right)}\left(k+1\right)=\left(x^{\left(1\right)}\left(1\right)-\frac{b}{a}\right)e^{-ak}+\frac{b}{a}. \end{array}$$
(4) The last step checks the prediction error between the predicted and actual values to determine the reliability of data analysis. The details are referred to Ju-Long [20]. Finally, we obtain two test ratings: the posteriori error *C* and small error possibility *P*. The precision grades are shown in Table 1.

### 2.2. Scenario Analysis

Scenario analysis, which is also called the scenario method, is a method that assumes a scenario will continue into the future and then forecasts the possible consequences of such a scenario. Scenarios are plausible stories about the future [22]. Scenario analysis is a qualitative method to investigate a result that is likely to appear under different tendencies. This can avoid the overestimation or underestimation of the variation. Given the superiority of scenario analysis in solving uncertainty and human dynamic role problems, scenario analysis has been widely used in strategic military purposes and certain areas' food supply capacity prediction [23–27].

In our research, we attempted to study the grain production capacity in Egypt by using a simplified scenario method. On the basis of previous studies on food supply capacity and Egyptian geography [28, 29], we selected three factors that mainly affect grain production in Egypt: cultivated area, percentage of grain area in cultivated area, and grain yield per unit (represented by *l*, *c*, and *y*); the relation between them is as follows:

(6) $$\begin{array}{c} F\left(l,c,y\right)=l\left(t\right)\times c\left(t\right)\times y\left(t\right), \end{array}$$

where *F* and *t* represent the grain production capacity and time. Grain yield denotes the harvest yield per unit area during one year; thus, grain yield is equal to the seeded area multiplied by multiple grain indices. On the basis of official statistics in *Egypt in Figures*  (2013), grain area is defined as cultivated areas in the three agricultural seasons and orchards; cultivated area denotes the area of land that is cultivated for agricultural crops without a repetition of the types of crops grown throughout the year [30]. We set the microvariation of the percentage of grain area in the cultivated area during the past 10 years to 85%. Therefore, we define different scenarios for cultivated area and grain yield. A detailed analysis is provided in subsequent sections of the paper.

### 2.3. Data Resource and Processing 

The data used in this study were selected from several sources. The production of main grains in Table 2 was obtained from *Egypt in Figures*  (2013) [30]. Data on long-term grain production, grain yield, grain area, and grain production per person were obtained mainly from FAOSTAT [31]. Because there is a small number of missing data, we use the data from Egypt in Figures (2011, 2012) to replace [30]. Microsoft Excel 2010 software was used to trim the data, SAS was used to process and calculate data, and Origin 8.0 was used to plot figures.

## 3. Grain Production Fluctuations in Egypt

### 3.1. Plant Structure in Egypt

Arable land in Egypt is limited and is mainly distributed around the Nile Valley and Nile Delta. The most widely plantedgrain cropsare rice, wheat, maize, and sorghum. The important economic crops are cotton, sugar cane, and sugar beet. Onion, tomato, and mango are the most popular horticulture plants in Egypt [32]. Wheat (32–48%) occupies the largest area among the grain crops, followed by maize (22–27%) and rice (16–29%).

### 3.2. Grain Production Fluctuating Law

**Figure 1:**
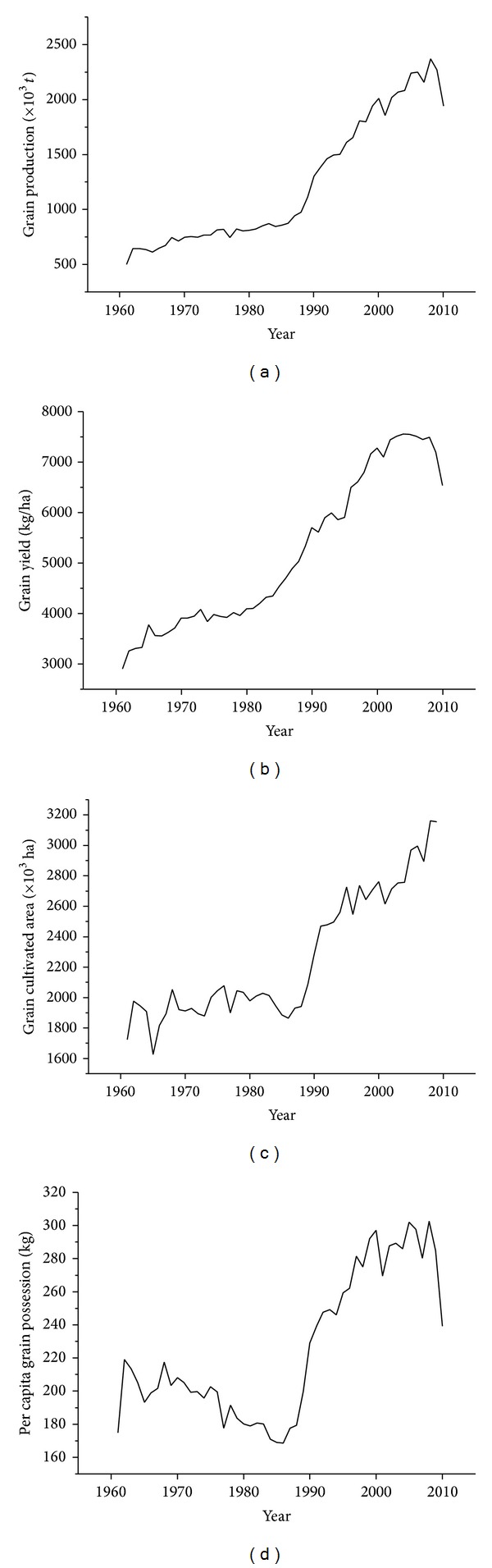
Change of grain production, grain yield, grain cultivated area, and per capita grain possession in Egypt (1961–2010). Source: FAO database and Egypt in Figures  2011–2013.

Figure 1(a) shows the changes of total grain production in Egypt from 1961 to 2010. The trend of grain production has generally increased in the past 50 years even though total grain production has decreased in the past 12 years. Grain production exhibited sustained growth from 1985 to 1997. The net increment reached 9.162 billion tonnes. The change curve could be divided into 3 stages by trends. The first stage (1961 to 1985) indicated an increase in production from 5 billion tons to 8.5 billion tonnes smoothly. The amount totally increased by 3.5 billion tonnes in 25 years with a step of 0.14 billion tonnes. The second stage occurred from 1986 to 2000. Grain production climbed considerably by 11.35 billion tonnes in this stage in 14 years; this increase in production is 133% more than the production in 1985. Governmental support, advanced technology, and grain production programs may have led to this production trend. The implementation of Mubarak's National Project in 1987 is another significant factor to the increase in grain production; this project grants graduates reclaimed land to encourage agricultural work [11]. The third stage (2001 to 2010) saw fluctuations in the appreciation of grain production. A sharp decrease occurred from 2008 to 2010; this reduction may have been caused by incremental extreme weather and natural disasters.

### 3.3. Changes of Unit Area Grain Yield

The unit area yield is a good reflection of the level of grain production. The changes of unit area grain yield in Egypt from 1961 to 2009 are clearly shown in Figure 1(b). The general tendency shows an increasing trend. The yield increased from 2905.7 kg/ha to 4347.4 kg/ha with 1.85% average annual growth rate during the first 23 years (1961 to 1983). The increment reached 1441.7 kg/ha, which is almost half of the yield in 1961. During the next 21 years (1984 to 2008), grain production stabilized and levelled off. The grain production peaked in 2004 at 7555.2 kg/ha, which is 73.79% and 160% more than the yield in 1984 and 1961, respectively. Agricultural techniques, for example, changes in fertilizer practice, irrigation and achievements in breeding high yielding varieties, and food policies, are the main reasons for the increasing of yield. However, the unit yield has decreased sharply since 2009. Many different factors may cause this decrease; low yield potential of new areas and degradation of formerly high yielding fields are major causes. Otherwise, extremes in weather conditions also have negative effects to yield. For a wide view on the variation trend, we speculated that the grain yield in Egypt encountered a choke point at 7500 kg/ha and the yield potential is limited and this limit was probably reached in 2010.

### 3.4. Changes in Grain-Cultivated Land Area

The area of grain-cultivated land reflects the development level of society and the economy and has an important impact on grain security.  Figure 1(c) shows the changes in the area of grain-cultivated land from 1961 in Egypt. This type of land has experienced a stationary phase with fluctuations from 1961 to 1985 and an increasing phase with fluctuations from 1986 to 2009. The trend exhibits a continuous substantial increase from 1986 to 1995 with a total increment of 0.86 million hectares. This rapid increase can be attributed to the government's support in reclaiming and using a large number of agricultural machinery in Egypt. The grain land area kept increasing from 1995 onward with slow growth and drastic fluctuations. The reasons for the characteristic changes may be as follows: (1) reduction in land that can be reclaimed and the expensive cost of residual land reclamation; (2) rat race between grain land and nonfood land because of increasing crop varieties; (3) degradation and salinization causing waste of a large amount of arable land [5]; (4) occupation of considerable amounts of land because of urbanization, thus causing a restriction and reduction in grain land. The research by Mohamed et al. shows that the area occupied by Egyptian cities has increased by 58% from 1972 to 1990 [33].

### 3.5. Per Capita Grain Possession

Per capita grain possession in Egypt is low according to the analysis of population and grain production. The per capita grain possession of Egypt exhibited a decreasing trend from 1961 to 1986 (Figure 1(d)). The grain production of Egypt in nine years was only 200 kg; the nadir appeared in 1986 with a grain production of 168.7 kg. Thereafter, the per capita grain possession kept increasing by 120 kg until 2000. From 2000 to 2010, the trend of grain production became volatile. The peak reached 302.5 kg in 2008. However, the number of grain possession decreased from a big range in 2001, 2007, 2009, and 2010. We can see that the per capita grain possession in Egypt is under 400 kg, which is not even half of the standard proposed by the United Nations, whether in 2008 (the “richest” year) or in other periods (Figure 1(d)).

### 3.6. Brief Summary

The above analysis shows that grain production, grain yield, and cultivated land grew slowly before 1986, except for the per capita grain possession, which decreased. By contrast, all the analysed indices increased rapidly and reached their respective peaks at around 2008. Economic/social factors, natural resources, and climatic conditions may have caused this change in characteristics. We calculated the correlation coefficient of the four indexes. The result shows that the correlation coefficient between two indexes is above 0.900 (*P* < 0.01), except between yield and per capita grain possession (i.e., 0.889 (*P* < 0.01)). The correlation coefficient between grain production and grain yield and between grain production and cultivated land reaches up to 0.984 and 0.978 (*P* < 0.01). The results show that grain yield and cultivated land significantly affect grain production.

## 4. Future Analysis of Population and Grain Supply in Egypt 

### 4.1. Egyptian Population Analysis and Prediction

**Figure 2:**
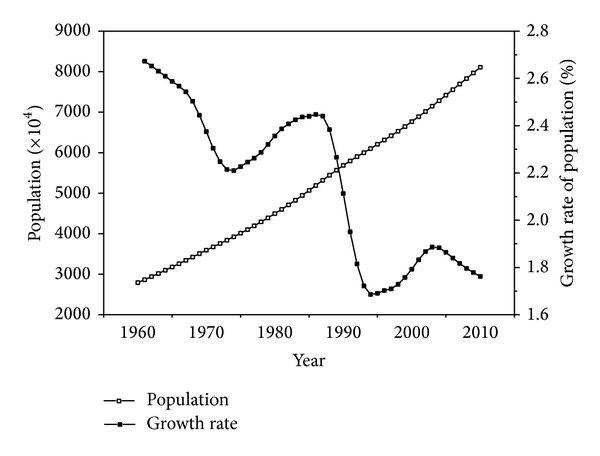
Population and growth rate in Egypt (1960–2010). Source: FAO database.

According to FAO statistical information (Figure 2), the size of the Egyptian population increased in the past half century. The total Egyptian population increased from 27.90 million in 1961 to 81 million in 2011. Net increment per year is more than 1 million. Figure 2 also indicates that the population change rate has decreased thrice and increased twice during the past 50 years. Although the overall trend of the population change rate has decreased by a large margin, the Egyptian population is always increasing. The slowdown of the population growth trend is only a facade.

We used the GM (1,1) model to analyse the Egyptian population from 1961 to 2011. The analytic process was conducted with SAS. The predicted value is shown in Figure 2. The optimal model of Egyptian population at time is

(7) $$\begin{array}{c} x\left(t+1\right)=1465882396.97e^{0.020526t}-1437979303.97. \end{array}$$

The analysis results show that the a posteriori error is *C* = 0.0647, small error possibility is *P* = 1, and the model precision is good and can be used for forecasting. Thereafter, we use this model to compute the future 20-year population of Egypt (Table 3).

The result shows that the Egyptian population will exceed 100 million by 2021 (Table 3) and will reach 123 million by 2030. The growth trend of the Egyptian population is nearly linear and grows faster with a growing base number. This increase in population will worsen the food supply problems in Egypt.

### 4.2. Analysis of Future Grain Production Capacity in Egypt

#### 4.2.1. Cultivated Area Acreage

Only 4% of total land can be used for agriculture activities. However, agricultural land has decreased significantly for several years because of urbanization and other reasons. This situation is a sign that agriculture in Egypt has reached a “grace period.” On the basis of analysis and previous studies, we established three scenarios for cultivated areas.

*High Scenario.* We suppose that the cultivated acreage will increase by 1.81% per year, similar to the annual increase from 2001 to 2005.However, for the limitation of arable land, 4 million ha was set as the maximum in the scenario calculations.

*Mid Scenario.* We suppose that the cultivated acreage will be equal to the cultivated acreage in 2011; the annual growth rate is zero.

*Low Scenario.* We suppose that the cultivated acreage will decrease by 0.94% per year, similar to the annual decrease from 2009 to 2011.

The predicted results in the main year are shown in Table 4.

In the high scenario, the cultivated acreage will keep increasing and reach 4 million before 2020 (Table 4). However, 4 million ha is the limit of arable land in Egypt. A continued increase is almost impossible. Under the low scenario, cultivated acreage will decrease slowly. Urbanization, soil salinization, and desertification are supposed to be the causes of land degeneration.

#### 4.2.2. Per Unit Area Grain Yield

The per unit area grain yield has shown increasing trends in waves for a long time. Although reductions begin to appear in 2004, particularly after 2008, the yield in 2010 is almost equal to the yield in 1996. Considering the abnormal changes after 2008, this result may be caused by some unconventional factors. Thus, we conducted a conservative estimation. The yield in 2008 (i.e., 7494.1 kg/ha) is used as the base value. We assume the following three scenarios for per unit area grain yield in the future in Egypt.

*High Scenario.* We assume that the grain yield will increase by 0.93% per year, similar to the annual gradient from 2000 to 2004.

*Mid Scenario.* We assume that the cultivated acreage will be equal to the cultivated acreage in 2008; the annual growth rate is zero.

*Low Scenario.* We assume that the grain yield will decrease by 0.46% per year, similar to the annual gradient from 2004 to 2007.

The predicted results in the main year are in Table 5.

The grain yield will increase by a relative large margin and will reach 8935.21 kg/ha in 2030 (Table 5). This may reflect a reduction in the demand of grain caused by the growing Egyptian population. However, the grain yield may fall below 70000.00 kg/ha after 2020 in low scenario, which will lead to a tense condition in grain supply.

#### 4.2.3. Scenario Prediction on Egyptian Grain Production Capacity

On the basis of the above analyses, we conducted a prediction on the future grain production capacity of Egypt (Table 6).

Grain production will keep increasing in the future if grain yield in high scenario and cultivated area acreage in any scenario or grain yield mid scenario and cultivated area acreage in high scenarios. The grain production will remain unchanged if the scenario is mid grain yield and mid cultivated area acreage (Table 6). Except for the mentioned scenarios, grain production will decrease on other scenario groups and the rate of grain production decrease will increase from high scenario to low scenario.

### 4.3. Per Capita Grain Supply Capacity in Egypt in the Future

We conducted a prediction of Egyptian population by using GM (1,1) model. By combining the grain production in different scenarios (Table 5) and the predicted population, we calculated the per capita grain possession in different future scenarios.

Among different scenarios, food supply can be basically satisfied (AR ≥ 80%) only in high cultivated areas and high grain yield scenarios before 2020, or in high cultivated areas and mid grain yield scenarios before 2015, under a standard of 400 kg per capita (Table 7). To ensure adequate food supply, both cultivated area and grain yield should be increased more. Under other circumstances, food supply will be insufficient. We used two extreme scenarios to estimate the future population carrying capacity of Egypt. We set the per capita grain possession to 400 kg, whereas multiple crop indices (180%) and the percentage of grain area in cultivated areas (85%) were unchanged. The population carrying capacity in 2030 is 89.35 million in both high yield and high cultivated area scenarios and is 51.45 million in the “double low” scenario. The corresponding grain production capacities are 3.57 million tons and 2.06 million tons.

## 5. Conclusion and Countermeasure

This study analysed future per capita grain possession in Egypt by exploring two main factors: population and grain production. The GM (1,1) model and scenario analysis method were used to conduct predictions. To show a comprehensive change in Egyptian grain production, a forward analysis was conducted on grain production, yield, cultivated area, and per capita grain possession in history.

During the period studied, the four analysed indices all exhibited a turning point around the year 1986. Slow growth or fluctuant growth occurred before 1986 and a sharp increase occurred thereafter. A series of policies, including Mubarak's National Project, are considered the main driving force of this change.

Population data from 1961 to 2011 were used to forecast the future population in Egypt by the GM (1,1) model, and the population will reach 123 million by 2030. The results show that the Egyptian population will continue to increase. The historical population growth rate appears as a wavy decreasing trend; however, the predicted data use a settled growth rate, which may cause errors in the analysis.

Scenario analysis of per capita grain possession shows a severe form of food insecurity in Egypt even though the current situation in Egypt is better than other African countries. Even in “double high” scenario (cultivated acreage increase by 1.81% and grain yield by 0.93% per year), the per capita grain possession will be 27% lacking under 400 kg per capita in 2030. A relatively high growth rate for cultivated areas and yields is needed if the country wants to have a sufficient grain supply. However, natural (e.g., extreme climate) and other factors (e.g., degradation and salinization of arable land) limit the arable land. Approximately 4 million ha is the choke point of cultivated land in Egypt. Thus, the only solution is to improve grain yield, which needs science and technology funding and economic and societal input.

Population carrying capacity is calculated based on two extreme scenarios that vastly differ. From 89.35 million to 51.45 million, the development patterns have a strong effect on future population carrying capacity. An increase in cultivated land and yield is needed to feed the increasing population of Egypt. Furthermore, population control measures should be implemented.

By combining the food security problem in Egypt with the research conclusions, we developed the following several development countermeasures.

1. Advanced agriculture technology should be to enhance per unit yield. The key points are to breed or import new and excellent crop strains to improve crop production capacity.
2. Strictly control urbanization and other uses of cultivated land and restrict degradation and salinization of arable land. Urbanization and industrialization are becoming a serious threat to arable land in Egypt. Therefore, government policies should address this threat. Arable land possession per capita in Egypt has always been <0.05 ha since 2000; this value is considerably less than the United States' at 5.88 ha and Europe's at 1.26 ha to 1.68 ha. If this predicament endures, Egypt will encounter an intense grain supply-demand problem. At the same time, measures that restrict degradation and salinization of cultivated land should be taken.
3. A suitable plan structural adjustment is needed. Rice has obvious high-yield advantages in Egypt compared with other crops. However, rice is only planted in restricted areas in Egypt because of limited natural resources. Wheat and maize are planted in considerable areas but have relatively low yield and quality. Thus, dry land should be transformed into paddy fields to increase the rice plantation area.
4. Formulating a reasonable crop production system is necessary to improve land utilization efficiency. The crop production index in Egypt is significantly high. Nevertheless, the use of unsuitable breeds and crops has resulted in low land output efficiency during crop in many places. Therefore, Egypt needs to improve its agricultural sector by importing suitable crop strains, developing technology, and implementing a reasonable strategy.

## Figures and Tables

**Table 1:** Precision grade table of GM (1, 1) model.

Small error possibility/*P*	Posteriori error/*C*	Predict precision grade
>0.95	<0.35	Good
>0.80	<0.5	Qualified
>0.70	<0.65	Approximately qualified
≤0.70	≥0.65	Unqualified

**Table 2:** Production and proportion of main grain crops in Egypt from 2007 to 2011.

Crops	2007			2008			2009			2010			2011		
	Prod.^a^ (10^3^ tons)	Area (10^3^ ha)	Prop. A^b^ (%)	Prod. (10^3^ tons)	Area (10^3^ ha)	Prop. A (%)	Prod. (10^3^ tons)	Area (10^3^ ha)	Prop. A (%)	Prod. (10^3^ tons)	Area (10^3^ ha)	Prop. A (%)	Prod. (10^3^ tons)	Area (10^3^ ha)	Prop. A (%)
Wheat	8274	1141	39.82	7977	848	32.74	8523	1335	44.95	7169	1273	47.12	8371	1281	46.61
Maize	6909	674	23.53	7401	690	26.63	7686	720	24.25	7183	710	26.28	6876	622	22.66
Rice	6755	703	24.53	7253	743	28.69	5520	575	19.36	4330	459	16.99	5675	592	21.54
Barley	136	103	3.59	149	76	2.95	149	95	3.20	117	37	1.37	122	36	1.30
Beans	257	99	3.45	352	80	3.08	553	105	3.55	340	85	3.14	255	61	2.23
Sorghum	887	146	5.09	867	153	5.90	781	140	4.71	702	138	5.11	839	155	5.66

Total	23218	2865	100	23999	2591	100	23212	2971	100	19841	2703	100	22138	2747	100

Data source: Egypt in Figures 2013; FAO database.

Note: ^a^Prod. is short for production.

^b^Prop. A is short for proportion of area.

**Table 3:** Prediction of the Egyptian population in 20 years using GM (1, 1) model.

Year	Predicted population (10^4^)
2014	8839.59
2015	9022.91
2016	9210.03
2017	9401.03
2018	9595.99
2019	9795.00
2020	9998.14
2021	10205.48
2022	10417.13
2023	10633.16
2024	10853.68
2025	11078.77
2026	11308.53
2027	11543.05
2028	11782.44
2029	12026.79
2030	12276.21
2031	12530.80
2032	12790.67
2033	13055.93

**Table 4:** Prediction of cultivated area acreage in Egypt.

Year	2015	2020	2025	2030
High scenario (ha)	3889459.78	4000000.00	4000000.00	4000000.00
Mid scenario (ha)	3620158.08	3620158.08	3620158.08	3620158.08
Low scenario (ha)	3490172.15	3334233.35	3185261.80	3042946.21

**Table 5:** Prediction of per unit area grain yield in Egypt (kg/ha).

Year	2015	2020	2025	2030
High scenario (kg/ha)	7776.79	8145.20	8531.07	8935.21
Mid scenario (kg/ha)	7494.10	7494.10	7494.10	7494.10
Low scenario (kg/ha)	7253.56	7086.48	6923.25	6763.79

**Table 6:** Scenario analysis of Egyptian grain production capacity in 20 years.

Scenarios		Grain production capacity (10^3^ tons)			
Cultivated area	Grain yield	2015	2020	2025	2030
High	High	**30247.51** ^ a^	**32580.80**	**34124.28**	**35740.84**
	Mid	**29148.00**	**29976.40**	**29976.40**	**29976.40**
	Low	28212.42	28345.93	27693.02	27055.15

Mid	High	**28153.21**	**29486.91**	**30883.82**	**32346.87**
	Mid	27129.83	27129.83	27129.83	27129.83
	Low	26259.02	25654.18	25063.28	24485.98

Low	High	**27142.34**	**27158.00**	**27173.69**	**27189.36**
	Mid	26155.70	24987.08	23870.67	22804.14
	Low	25316.16	23627.98	22052.38	20581.84

Note: ^a^figures in bold indicated the increasing scenarios of grain production capacity.

**Table 7:** Per capita grain possession and assurance rate^a^ in different scenario groups of Egypt.

Year		2015		2020		2025		2030	
Cultivated area	Grain yield	Po^b^ (kg/person)	AR^c^ (%)	Po (kg/person)	AR (%)	Po (kg/person)	AR (%)	Po (kg/person)	AR (%)
High	High	**335.23** ^ d^	**0.84**	**325.87**	**0.81**	**308.02**	**0.77**	**291.14**	**0.73**
	Mid	323.04	0.81	299.82	0.75	270.58	0.68	244.18	0.61
	Low	312.68	0.78	283.51	0.71	249.96	0.62	220.39	0.55

Mid	High	312.02	0.78	294.92	0.74	278.77	0.70	263.49	0.66
	Mid	300.68	0.75	271.35	0.68	244.88	0.61	221.00	0.55
	Low	291.03	0.73	256.59	0.64	226.23	0.57	199.46	0.50

Low	High	300.82	0.75	271.63	0.68	245.28	0.61	221.48	0.55
	Mid	289.88	0.72	249.92	0.62	215.46	0.54	185.76	0.46
	Low	**280.58**	**0.70**	**236.32**	**0.59**	**199.05**	**0.50**	**167.66**	**0.42**

Note: ^a^400 kg per capita per year was regarded as a threshold for food security. Many scholars considered 400 kg per capita as assumed to represent a threshold of cereal supply for sustained economic growth.

^b^Po is short for per capita grain possession.

^c^AR is short for assurance rate, which is the percentage of per capita grain possession on 400 kg [34].

^d^Figures in bold indicated the extreme scenarios of per capita grain possession.
